# DSE promotes aggressive glioma cell phenotypes by enhancing HB-EGF/ErbB signaling

**DOI:** 10.1371/journal.pone.0198364

**Published:** 2018-06-04

**Authors:** Wen-Chieh Liao, Chih-Kai Liao, You-Huan Tsai, To-Jung Tseng, Li-Ching Chuang, Chyn-Tair Lan, Hung-Ming Chang, Chiung-Hui Liu

**Affiliations:** 1 Department of Anatomy, Faculty of Medicine, Chung Shan Medical University, Taichung, Taiwan; 2 Department of Medical Education, Chung Shan Medical University Hospital, Taichung, Taiwan; 3 Department of Anatomy and Cell Biology, School of Medicine, College of Medicine, Taipei Medical University, Taipei, Taiwan; Northern University, UNITED STATES

## Abstract

Remodeling of the extracellular matrix (ECM) in the tumor microenvironment promotes glioma progression. Chondroitin sulfate (CS) proteoglycans appear in the ECM and on the cell surface, and can be catalyzed by dermatan sulfate epimerase to form chondroitin sulfate/dermatan sulfate (CS/DS) hybrid chains. Dermatan sulfate epimerase 1 (DSE) is overexpressed in many types of cancer, and CS/DS chains mediate several growth factor signals. However, the role of DSE in gliomas has never been explored. In the present study, we determined the expression of DSE in gliomas by consulting a public database and conducting immunohistochemistry on a tissue array. Our investigation revealed that DSE was upregulated in gliomas compared with normal brain tissue. Furthermore, high DSE expression was associated with advanced tumor grade and poor survival. We found high DSE expression in several glioblastoma cell lines, and DSE expression directly mediated DS chain formation in glioblastoma cells. Knockdown of DSE suppressed the proliferation, migration, and invasion of glioblastoma cells. In contrast, overexpression of DSE in GL261 cells enhanced these malignant phenotypes and *in vivo* tumor growth. Interestingly, we found that DSE selectively regulated heparin-binding EGF-like growth factor (HB-EGF)-induced signaling in glioblastoma cells. Inhibiting epidermal growth factor receptor (EGFR) and ErbB2 with afatinib suppressed DSE-enhanced malignant phenotypes, establishing the critical role of the ErbB pathway in regulating the effects of DSE expression. This evidence indicates that upregulation of DSE in gliomas contributes to malignant behavior in cancer cells. We provide novel insight into the significance of DS chains in ErbB signaling and glioma pathogenesis.

## Introduction

High grade gliomas, including grade III anaplastic astrocytomas and grade IV glioblastomas, are among the most aggressive human cancers. They are the third greatest cause of cancer death in people under the age of 35 worldwide [1]. Currently, glioblastomas are incurable. The average survival rate of glioblastoma is less than 2 years, even in patients who have received standard surgical resection followed by radiation and chemotherapy, or enrollment in a clinical trial. The high mortality of this disease is mainly attributable to the limited treatment options, and the almost inevitable recurrence after surgical care [2, 3]. In this regard, elucidation of the precise molecular mechanisms underlying glioma progression is crucial for developing new treatments of this fatal disease.

The aberrant expression of extracellular matrix (ECM) proteins and an abnormal glycan composition in the tumor microenvironment are hallmarks of all types of cancer [4, 5]. In contrast to other organs, the ECM of the central nervous system (CNS) stroma comprises abundant glycosaminoglycans (GAGs) and proteoglycans (PGs), instead of collagens or laminins [6]. GAGs are composed of unbranched polysaccharide chains such as heparan sulfate (HS), chondroitin sulfate (CS), and dermatan sulfate (DS). They can exist as free chains or may be covalently linked to a core protein, as in chondroitin sulfate proteoglycan (CSPG) and heparan sulfate proteoglycan (HSPG). CS chains are composed of repeating glucuronic acid/N-acetylgalactosamine (GlcA-GalNAc) blocks with complex sulfation at various positions. In certain tissues, C5 epimerase converts GlcA to iduronic acid (IdoA) within the CS chains. These IdoA-GalNAc units constitute dermatan sulfate, and are usually designated as CS/DS chains to demonstrate their hybrid nature [7–9].

In the CNS, CS chains are one of the major components of glial scars, which prevent nerve regeneration. The use of chondroitinase ABC or CSPG inhibitor to eliminate CS chain deposits in the lesioned dorsal columns promotes functional recovery from spinal injuries [10, 11]. Studies have shown that the levels of several CSPGs increase significantly in the ECM of gliomas [8, 12]. The degradation of CSPG in gliomas by oncolytic viruses expressing bacterial chondroitinase ABC promotes the spread and anti-tumor efficacy of the viruses [13]. Moreover, a recent study revealed that cleaving CS chains increases the availability of temozolomide and its effectiveness on glioma cells [14]. These studies highlight the importance and therapeutic targeting potential of CS chains in the CNS.

As well as presenting a barrier, it is thought that heterogeneous GAG structures interact with various growth factors, proteases, cytokines, and adhesion molecules on tumor cells, resulting in the regulation of cell growth, differentiation, angiogenesis, and cell migration in various types of cancer [15–17]. For instance, increased levels of CS chains in hepatocellular carcinoma cells may promote aggressive phenotypes, and enhance hedgehog signaling [18]. CS chains in melanoma cells can enhance MMP2 activation, and promote angiogenesis, proliferation, and cell invasion [19, 20]. Overexpression of DS epimerase 1 in squamous cell carcinoma increases levels of DS chains in cancer tissue, which can mediate hepatocyte growth factor (HGF) signaling [21]. Moreover, a unique onco-fetal CS modification was recently found, which could be used as a diagnostic marker or a drug target in many types of cancer [22]. These findings point to the oncogenic function of aberrant CS/DS chains in many types of tumor cells. It is known that CS/DS hybrid chains from brain tissue also bind to several growth factors and neurotrophic factors, suggesting their essential role in tissue morphogenesis [9, 23, 24]. However, their biological functions and modifications in glioma progression remain largely unknown.

## Materials and methods

### Cell culture

Human glioma cell lines, GBM8401, GBM8901, and DBTRG-05MG were purchased from Bioresource Collection and Research Center in the year 2014 (Hsinchu, Taiwan). Human glioma cell lines, A172, LN18, U118, and U251-MG, and mouse glioma cells, GL261, were kindly provided by Dr. Wei KC (Chang Gung Memorial Hospital). Cells were cultured in DMEM containing 10% FBS in 5% CO2 at 37°C.

### Reagents and antibodies

Recombinant HB-EGF, NRG1, and EGF protein were purchased from PeproTech. Full length DSE-pcDNA3.1 plasmid was purchased from GeneScript. Two pLKO.1/DSE-shRNA plasmids (DSE sh1, 5’- CAGAAAGAACTACCCATAGAT -3’; DSE sh2, 5’- CAGAAAGAACTACCCATAGAT -3’) and nontargeting pLKO.1 plasmids were purchased from National RNAi Core Facility (Academia Sinica, Taipei, Taiwan). ON-TARGETplus SMARTpool siRNA against human DSE was purchase from Dharmacon. CCK8 reagent was purchased from Sigma-Aldrich. Rabbit polyclonal anti-DSE antibody was purchased from Sigma-Aldrich. The immunogen of this DSE antibody is from human DSE sequence which is 87% identical to mouse Dse sequence. Antibodies against p-AKT, p-ERK1/2, ERK1/2, p-EGFR (Y1068), EGFR, and p-ErbB2(Y1248) were purchased from Cell Signaling Technology. Antibodies against total AKT and Actin were purchased from GeneTex, Inc. Antibody against ErbB2 was purchased from Santa Cruz Biotechnology. FITC conjugated anti-rabbit IgG was purchased from Invitrogen. HRP conjugated-DS binding protein was purchased from Lifespan Technologies. The dual EGFR/HER2 inhibitor, Afatinib, was purchased from MedChemExpress.

### Tissue array and immunohistochemistry

Paraffin-embedded human glioma tissue microarrays with five normal brain tissue were purchased from Shanghai Outdo Biotech and Pantomics, Inc. UltraVision Quanto Detection System (Thermo Fisher Scientific Inc.) was used for immunohistochemistry. Arrays were incubated with anti-DSE antibody (1:100) in 5% bovine serum albumin/PBS 16 hours at 4°C. The specific immunostaining was visualized with 3,3-diaminobenzidine and counterstained with hematoxylin (Sigma). Images were obtained by TissueFAX Plus Cytometer. The staining index was calculated as previous descripted [16]. A final score ≥ 6 were be considered high expression.

### Transfection

For overexpression experiments, empty pcDNA3.1 and DSE-pcDNA3.1 plasmids were transfected to GL261 cells using Lipofectamine 2000 (Invitrogen). The transfected cells were selected with 600 μg/mL of G418 for 14 days. For transient knockdown of DSE, cells were transfected with 20 nmol of siRNA using Lipofectamine RNAiMAX (Invitrogen) for 48 hours. The short hairpin RNA (shRNA) plasmids were transfected to U118 cells using Gene Pulser Xcell electroporation system (960uF, 200V) and selected with 1000 ng/mL of puromycin for 10 days. Overexpression and knockdown of DSE in transfectants were confirmed by Western blotting.

### Western blotting and DS chains blotting

Western blotting was carried out as reported previously [25]. To analyze DS chains on proteoglycans, 80 μg of total protein lysate were separated by 10% SDS-PAGE and transferred to PVDF membrane. To simply the measurement of the changes to the DS chains in the cell lysate, we used horseradish peroxidase (HRP)-conjugated DS binding protein on western blots, and visualized by ECL. Total protein was measured by stain-free technology (Bio-Rad).

### Immunofluorescence microscopy and confocal microscopy

Cells were plated onto coverslips in 24 well culture plates for 24 hours. For DSE staining, cells were fixed in 4% paraformaldehyde and stained with anti-DSE antibody and FITC-conjugated anti-rabbit IgG. Hoechst 33342 was used for nuclear staining. Images were captured by ZEISS Axio Imager A2 microscope. The confocal images of immunofluorescence staining were captured with a confocal microscope, Zeiss LSM 510 META. Five serial scan images within a total thickness of 3.2 μm were obtained for each field.

### Dermatan sulfate ELISA

Double antibody ELISA kit for detecting Dermatan sulfate was purchased from MyBioSource (MBS266153). The assay was performed according to manufacturer’s protocol. 0.3 mg of total cell protein lysate in 100 μl lysis buffer was used for each test, and the specimens were triplicate.

### CCK-8 assay and colony formation

Cells (2 × 10^3^) were seeded into 96-well plates with culture medium 16 hours before the experiments. Cell viability was analyzed by CCK-8 reagent at 0, 24, 48, and 72 hours. Four wells per group for each time point were measured following manufacturer’s protocol. The experiments were repeated for three times, and relative fold changes of OD 450 nm were shown.

For anchorage-dependent colony formation assay, 500 cells were seeded in 6-well plates. Colonies were stained by crystal violet and counted after two weeks incubation.

### Cell migration and invasion assay

Transwell inserts with uncoated porous filters (pore size 8 μm) were used to evaluate cell migration, and Matrigel (BD Biosciences) coated porous filters were used to measure cell invasion. 2×10^4^ cells in serum-free DMEM were seeded into inserts, DMEM containing 10% FBS was added in lower part of the inserts for 16 hours incubation. Independent experiments were repeated for at least three times. Average number of cells per microscopic field was shown.

### Mouse model for *in vivo* tumor growth

8 weeks male C57BL/6 mice were purchased from National Laboratory Animal Center (Tainan, Taiwan). 2.5 × 10^6^ of GL261 mock transfectants and DSE transfectants were injected into right flank subcutaneously (n = 6). Tumor volumes were monitored at day 0, day 4, day 8, and day 14. Excised tumors were weighed and analyzed by Western blotting.

All animal experiments in this study were reviewed and approved by the Institutional Animal Care and Use Committee (IACUC) of Chung Shan Medical University Experimental Animal Center.

### Statistical analysis

All data analysis was performed using GraphPad Prism 6. One way ANOVA multiple comparisons were used for analyzing *DSE* gene expression in different glioma subtypes. Student t test was used for statistical analyses. Two-sided Fisher exact test was used for comparisons between DSE expression and clinicopathologic features of glioma tissue array. P < 0.05 was considered statistically significant.

## Results

### Dermatan sulfate epimerase (DSE) is frequently upregulated in gliomas, and correlates with high tumor grade and poor survival

**Fig 1:**
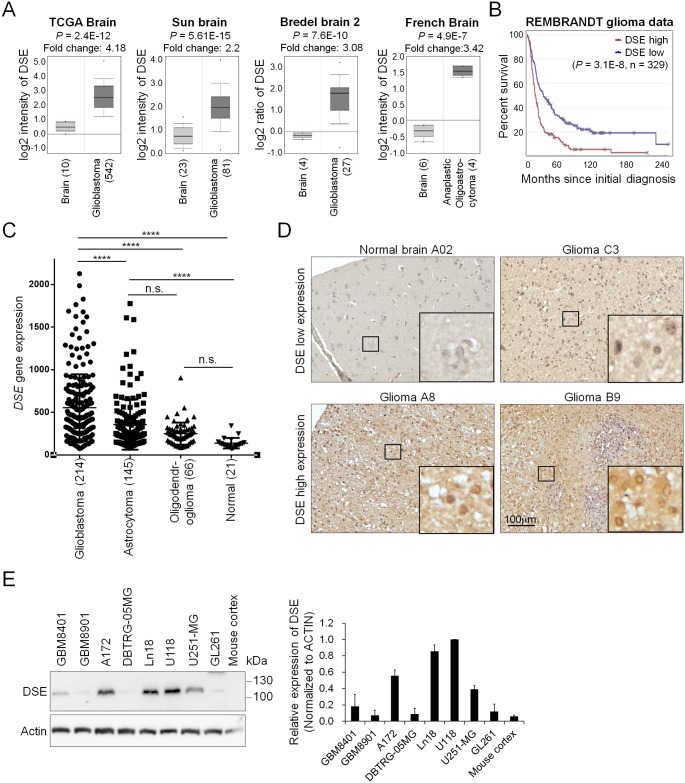
Dermatan sulfate epimerase 1 (DSE) is frequently upregulated in human gliomas. (A) Expression of DSE in the ONCOMINE cancer microarray database. Four independent representative datasets showed that DSE is significantly upregulated in glioma tissue. (B) High expression of DSE is associated with worse overall survival in glioma patients. The high and low expression groups are divided by median expression levels of DSE in 329 cases. (C) Comparison of *DSE* gene expression in glioma subtypes and normal brain tissue. ****P<0.0001, n.s. = not statistically significant. These data are from the REMBRANDT database (http://www.betastasis.com/glioma/rembrandt/). (D) Immunohistochemistry of DSE on a tissue array comprising 77 primary glioma samples and 5 normal human brain tissue samples. All sections were counterstained with hematoxylin. Representative images of normal brain tissue; one DSE low expression case (upper panel), and two DSE high expression cases (lower panel) are shown. Amplified images are shown at the bottom right of each image. Scale bars, 100 μm. (E) Expression of DSE in glioma cell lines and mouse brain tissue. Protein expression was analyzed by western blotting. Actin was used as an internal control. Relative expression levels in U118 cells from three independent blots are shown at the right.

**Fig 2:**
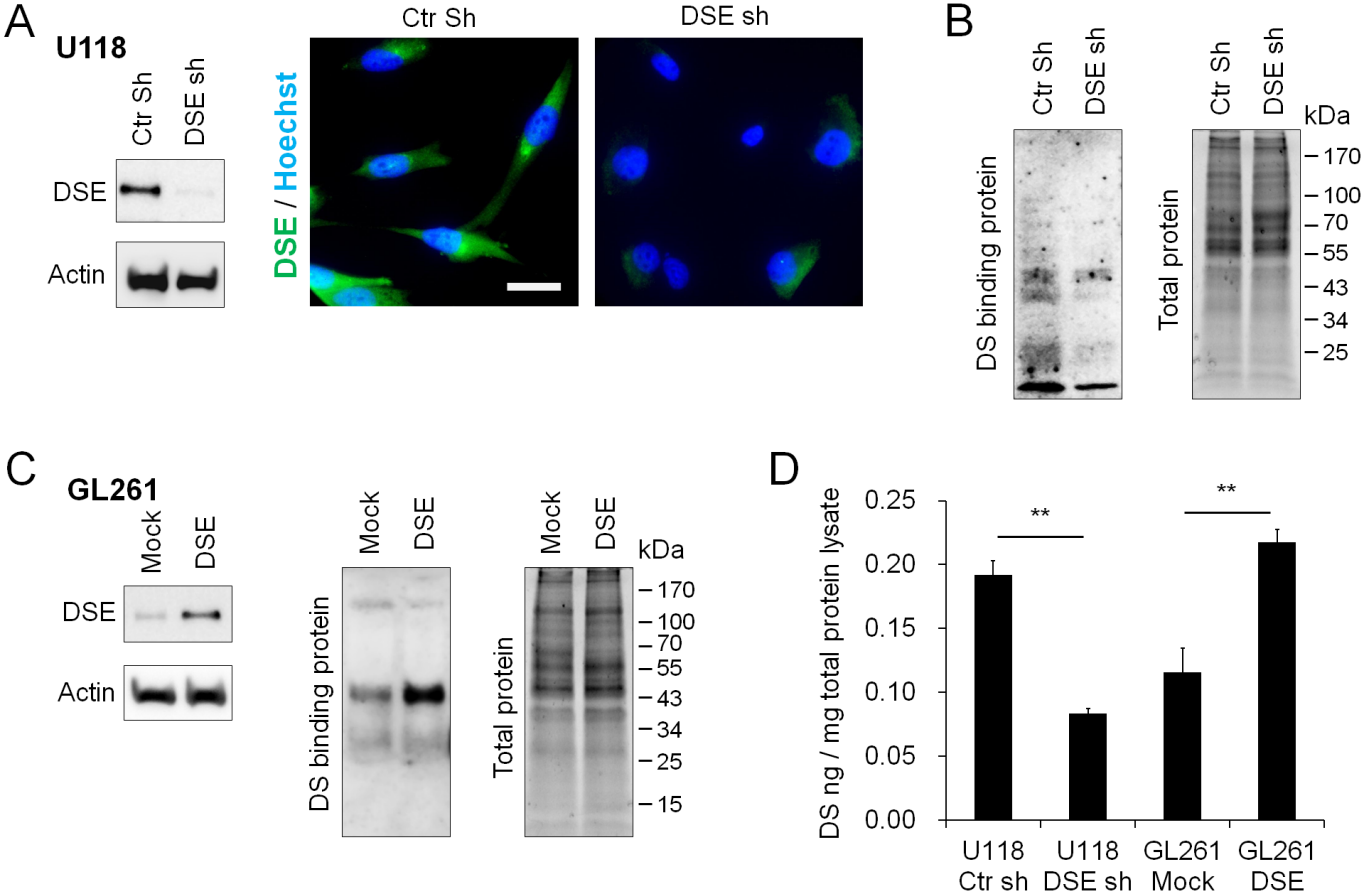
Dermatan sulfate epimerase 1 (DSE) regulates dermatan sulfate formation in glioma cells. (A) Stable knockdown of DSE in U118 cells. U118 cells were stably transfected with control short hairpin RNA (shRNA) (Ctr sh) or DSE-shRNA (DSE sh). The protein expression levels of DSE were analyzed by Western blotting. Immunofluorescence microscopy showed decrease of DSE (green) in stable knockdown cells. Nuclei were counterstained with Hoechst (blue). (B) Blotting of DS chains on proteoglycans was evaluated by horseradish peroxidase (HRP)-conjugated DS-binding protein. Total protein is shown on the right as loading control. (C) Overexpression of DSE in GL261 cells increased DS chain formation. GL261 cells were stably transfected with empty vectors (mock) or DSE-expressing plasmids (DSE). (D) Quantify DS in total protein lysate by DS ELISA assay. Average among of DS in cell lysate was shown. **P < 0.01.

The biosynthesis of the iduronic acid in CS/DS chains is mediated by two epimerases: DS epimerase 1 (encoded by the *DSE* gene) and DS epimerase 2 (encoded by the *DSEL* gene) [26]. We first researched the expression of these two epimerases in the ONCOMINE database [27]. Compared with normal brain tissue, 6 out of 26 independent datasets indicated that only *DSE* is significantly upregulated in brain tumors, especially in grade IV glioblastomas (Fig 1A). Furthermore, a search of the REpository for Molecular BRAin Neoplasia DaTa (REMBRANDT) database revealed that high expression of *DSE* is associated with worse overall survival in glioma patients (n = 329), whereas *DSEL* expression does not correlate with patient outcomes (S1 Fig). In addition, *DSE* expression in glioblastoma is significantly higher than that in astrocytoma, oligodendroglioma, and normal brain tissue (Fig 2B). We conducted an immunohistochemical investigation of DSE protein expression in a glioma tissue array containing 77 primary glioma tissue samples and 5 non-tumor brain tissue samples. The immunohistochemical investigation revealed that DSE was expressed in the paranuclear and perinuclear cytoplasm of certain glioma tissue (Fig 1C). No obvious DSE staining was found in the normal brain tissue. To assess the correlation with clinicopathologic characteristics, DSE expression was classified into two levels: low (staining index < 6) and high (staining index ≥ 6). Our data revealed that DSE was highly expressed in 73% (56/77) of the glioma tumors, whereas all the normal brain tissues exhibited very weak DSE expression (two-sided Fisher exact test, P = 0.0024) (Fig 1C and Table 1). Furthermore, high DSE expression in glioma tissue was associated with the worst histologic grade (Table 1). These results are consistent with those reported for *DSE* gene expression in the public databases. In an examination of DSE protein levels in human glioblastoma cell lines, mouse glioblastoma GL261 cells, and mouse brain cortex tissue, we found that 5 out of 7 human glioblastoma cell lines expressed relative high levels of DSE. This anti-DSE antibody also detected endogenous mouse Dse in GL261 cells, and expression of Dse in mouse cortex tissue is very low (Fig 1D). These data suggest that DSE is frequently upregulated in glioma patients, and its expression is correlated with the worst histology grade and poor overall survival.

**Table 1:** Correlation between DSE expression in glioma tissue and clinicopathological features.

Factor		DSE expression		*P* value (Two-sided Fisher’s exact test)
		Low	High	
Tissue types	Non-tumor	5	0	0.0024*
	Tumor	21	56	
Sex^#^	Male	8	27	0.785
	Female	9	25	
Age^#^	< 55 years	10	20	0.167
	≥ 55 years	7	32	
Tumor stage	Grade I–III^$^	14	19	0.019*
	Grade IV (GBM)	7	37	

*P < 0.05 was considered as statistically significant.

^#^Eight patients’ sex and age were not provided.

^$^Astrocytoma and Oligodendroglioma.

### DSE mediates dermatan sulfate formation in glioma cells

We knocked down DSE in U118 cells and overexpressed it in GL261 cells to investigate the functions of DSE in DS formation. Stable DSE knockdown clones of U118 cells were established by transfection with two short hairpin RNA (shRNA) plasmids (S2 Fig). The DSE knockdown clones were pooled together for further investigation. Western blots and immunofluorescence staining demonstrated that DSE decreased in knockdown cells (Fig 2A). Confocal microscopy was used to reveal subcellular localization of DSE, which mainly expressed in paranuclear and perinuclear cytoplasm (S3 Fig). In addition, we found that DSE knockdown reduced the binding of the DS-binding protein to proteoglycans in the U118 cells (Fig 2B), whereas overexpression of DSE enhanced the DS-binding protein signals in the GL261 cells (Fig 2C). Furthermore, the DS ELISA assay showed that DSE knockdown decreased DS in cell lysates, whereas DSE overexpression increased DS quantity in cell lysate (Fig 2D). These results indicate that DSE mediates the formation of DS chains on several proteoglycans in glioma cells.

### DSE regulates the proliferation, migration, invasion, and tumor growth of glioma cells

**Fig 3:**
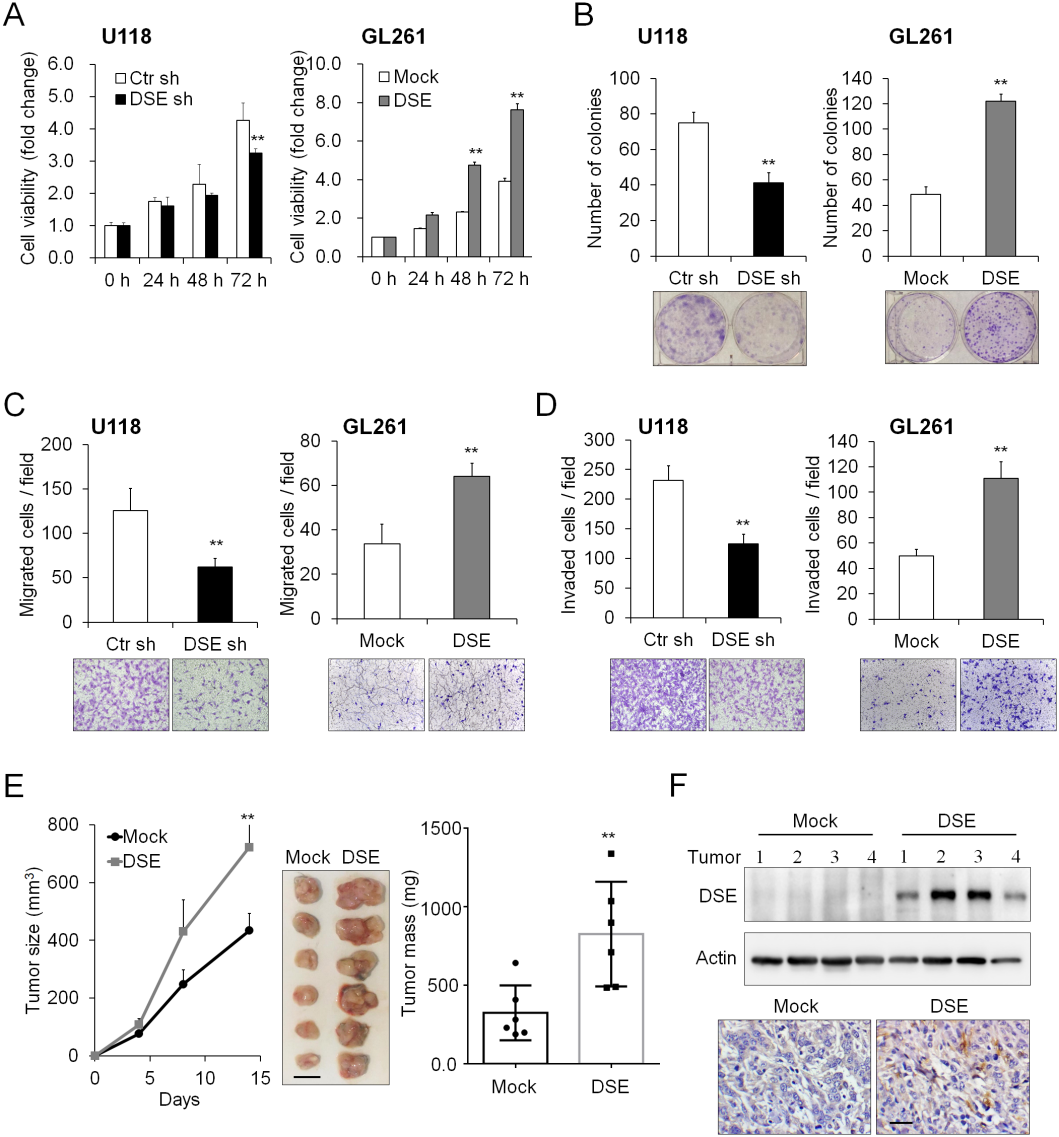
Dermatan sulfate epimerase 1 (DSE) regulates malignant phenotypes in glioma cells. (A) DSE modulated cell viability *in vitro*. The cell viability of U118 and GL261 cells was determined using a CCK-8 assay at the indicted time-points. Data represent means ± SD from three independent experiments. *P < 0.05; **P < 0.01. (B) Effects of DSE on anchorage-dependent colony formation. Representative images of colonies are shown at the bottom. Results are presented as the mean ± SD from three independent experiments. **P < 0.01. (C) Effects of DSE on Transwell cell migration, and (D) Matrigel invasion. Representative images are shown at the bottom. All results are represented as means ± SD from three independent experiments. **P < 0.01. (E) DSE enhanced tumor growth *in vivo*. GL261 transfectants were subcutaneously injected into C57BL/6 mice. The size of the tumors was measured at the indicated time-points, and is represented as the mean ± SD. Tumors were excised and weighted on the 14th day. **P < 0.01, n = 6. Scale bars, 0.5 cm. (F) Expression of DSE in excised tumors. The protein lysate was analyzed by western blotting (top). Actin was used as a loading control. Immunohistochemistry of DSE in tumors (bottom). Representative images are shown. Scale bars, 50 μm.

We investigated the effects of DSE on malignant phenotypes, cell viability, colony formation, and cell migration and invasion in DSE-knockdown and DSE-overexpressed clones. Our data demonstrated that DSE knockdown significantly suppressed cell viability in U118 cells and Ln18 cells, whereas overexpression of DSE enhanced cell viability (Fig 3A and S4 Fig). Consistently, DSE knockdown reduced the number of anchorage-dependent colonies, whereas overexpression of DSE dramatically increased the number of colonies (Fig 3B). Furthermore, DSE knockdown significantly reduced FBS-induced migration and invasion of U118 cells, whereas overexpression of DSE significantly promoted migration and invasion of GL261 cells (Fig 3C and 3D). To investigate the effects of DSE on tumor growth *in vivo*, we subcutaneously transplanted mock and DSE-overexpressed GL261 cells into mice. Our results showed that overexpression of DSE significantly enhanced the volume and weight of the tumors (Fig 3E). Western blots and the immunohistochemistry of the excised tumors confirmed the expression of DSE in tumor tissue (Fig 3F). These data indicate that DSE can modulate the cell viability, colony formation, cell migration and invasion, and *in vivo* tumor growth of glioma cells.

### DSE mediates heparin-binding EGF-like growth factor (HB-EGF)/ErbB signaling in glioma cells

**Fig 4:**
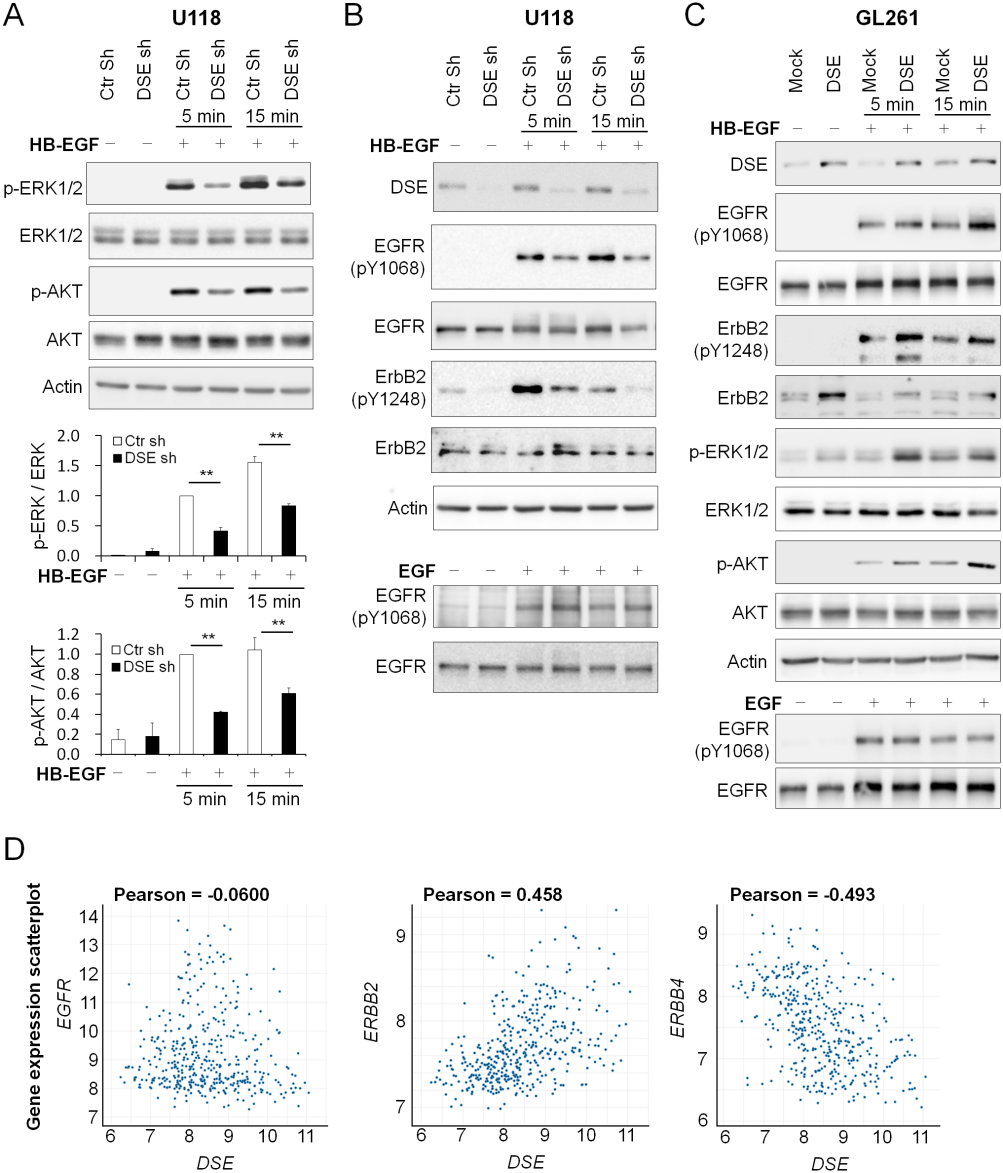
Dermatan sulfate epimerase 1 (DSE) modulates heparin-binding EGF-like growth factor (HB-EGF)/ErbB signaling in glioma cells. (A) Knockdown of DSE suppressed HB-EGF-induced downstream signaling. U118 transfectants were treated without (−)/with (+) HB-EGF for 5 and 15 min. Phosphorylation levels of ERK, AKT, total ERK, and AKT were measured by western blotting. Signals were quantified by Image J, and represented as means ± SD from three independent experiments. **P < 0.01 (B) Knockdown of DSE suppressed epidermal growth factor receptor (EGFR) and ErbB2 activation. The protein expression and phosphorylation of EGFR and ErbB2 were analyzed by western blotting with the indicated antibodies. Actin was used as a loading control. (C) Overexpression of DSE enhanced HB-EGF-induced signaling. GL261 transfectants were treated without (−)/with (+) HB-EGF or EGF. Cell lysates were analyzed by western blotting with various antibodies, as indicated. (D) The correlation of *DSE* expression with *EGFR*, *ERBB2*, and *ERBB4*. Expression of *DSE* and *ERBB2* were positively correlated in glioma patients. Data were analyzed using the REMBRANDT database (http://www.betastasis.com/glioma/rembrandt/).

Aberrations in the ErbB pathway are the most common causes of alterations in human gliomas [28, 29], and CS/DS hybrid chains have the ability to interact with several growth factors [7]. We next determined whether DSE regulates the ErbB signaling pathways in glioma cells. We treated glioma cells with the following recombinant ErbB family ligands: EGF, NRG1, and HB-EGF protein. We found that DSE knockdown in U118 cells selectively suppressed HB-EGF-triggered downstream ERK and AKT activation, whereas no obvious differences were observed following NRG1 and EGF treatment (Fig 4A and S5 Fig). HB-EGF can activate epidermal growth factor receptor (EGFR) homodimers or EGFR/ErbB2 heterodimers. We next investigated the phosphorylation of EGFR (Y1068) and ErbB2 (Y1248). DSE knockdown in U118 cells reduced HB-EGF-induced EGFR and ErbB2 activation, whereas it did not significantly alter EGF-induced EGFR activity (Fig 4B). In contrast, the overexpression of DSE in GL261 cells enhanced HB-EGF-induced EGFR and ErbB2 phosphorylation, as well as downstream ERK and AKT signaling, whereas EGF-triggered EGFR activation was still not affected (Fig 4C). Interestingly, we observed that overexpression of DSE in GL261 cells significantly increased the total protein level of ErbB2 but not of EGFR, and DSE knockdown in the U118 cells attenuated total ErbB2 expression (Fig 4B and 4C). We searched for correlation between DSE and the ErbB family in the REMBRANDT database, and found that the *DSE* gene was mildly positive associated with *ErbB2* expression and mildly negative correlated with *ErbB4* (Fig 4D). These results imply that DSE could involve the expression of *ErbB2* and *ErbB4* in glioma cells.

**Fig 5:**
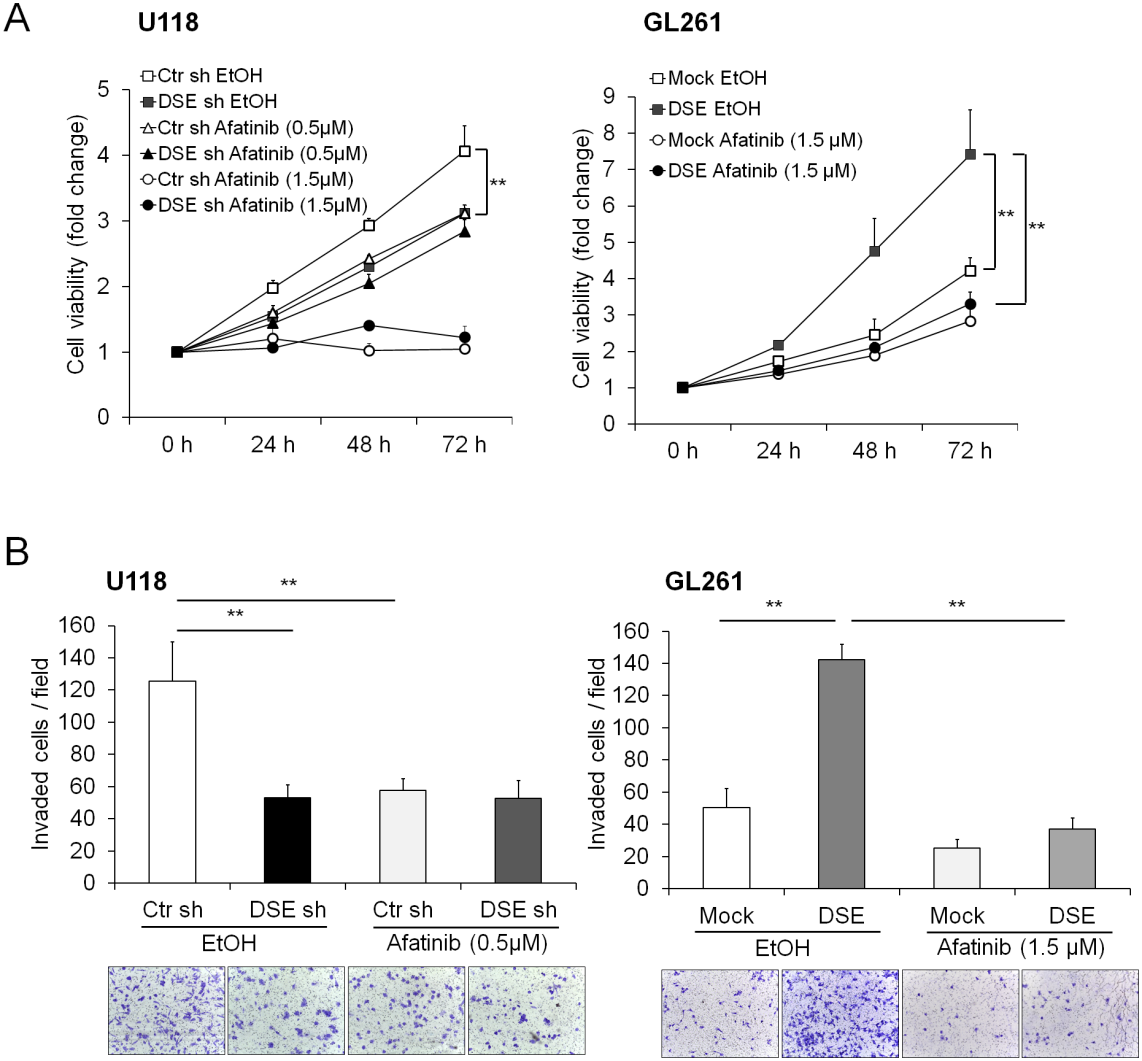
Effects of afatinib on dermatan sulfate epimerase 1 (DSE)-mediated malignant phenotypes. U118 and GL261 transfectants were treated with ethanol (EtOH, solvent control) or the indicated concentrations of afatinib. (A) Cell viability was measured using a CCK8 assay. (B) Cell invasion was analyzed using a Transwell assay. All results are represented as means ± SD from three independent experiments. **P < 0.01.

We treated glioma cells with an irreversible dual EGFR/ErbB2 inhibitor (afatinib) to evaluate the effects of the EGFR and ErbB2 pathways on DSE-regulated malignant phenotypes. We found that low concentrations of afatinib (0.5 μM) significantly suppressed the cell viability of the control shRNA-transfected U118 cells, but not that of the DSE-knockdown U118 cells. Furthermore, DSE-enhanced cell viability was suppressed by treatment with afatinib in GL261 cells (Fig 5A). A transwell invasion assay revealed that the solvent control (100% of EtOH) attenuated cell invasion in both control and DSE-knockdown cells, and afatinib treatment suppressed control cells invasion but did not further suppress cell invasion in DSE-knockdown U118 cells (Figs 5B and 3D). In addition, afatinib treatment suppressed DSE-enhanced cell invasion in GL261 cells (Fig 5B). Although we cannot exclude that other pathways may involve in DSE-mediated phenotypes, blocking EGFR/ErbB2 signaling by afatinib can inhibit DSE-induced malignant phenotypes in glioma cells.

## Discussion

The present study showed that DSE is frequently upregulated in human glioma tissue and cell lines, and DSE upregulation in glioma tissue is associated with a worse tumor grade and poor overall survival. The formation of DS on proteoglycans is regulated by DSE expression in glioma cells. DSE knockdown suppresses malignant phenotypes, whereas DSE overexpression enhances glioma cell malignancy, both *in vitro* and *in vivo*. Mechanically, DSE modulates HB-EGF-induced EGFR/ErbB2 activity and downstream signaling, whereas DSE-induced cell viability and invasion are hindered by EGFR/ErbB2 inhibitors. For the first time, the present study has shown that dermatan sulfate epimerase (DSE) is capable of regulating glioma cell malignancy *in vitro* and *in vivo*, and the ErbB pathway is involved in this process. Our findings provide a novel insight into the biological functions of DS chains in the pathogenesis of glioma, and may be useful in the development of a selective marker for glioma treatment.

In the past, DSE was considered a cancer cell antigen, and was called squamous cell carcinoma antigen recognized by T-cells 2 (SART2); it was supposedly highly expressed in most squamous cell carcinomas from various organs, including a subpopulation of colorectal carcinoma [30, 31]. Our search of the ONCOMINE database showed that 6 out of 26 analyses (23%) of brain and CNS cancers revealed a greater than two-fold increase in the level of DSE, compared with normal tissue. Among the 20 cancer types classified in ONCOMINE, brain and CNS cancer have the highest ratio of DSE increase, and the 6 datasets mentioned above all refer to gliomas and glioblastomas. The results of the immunohistochemical glioma tissue array investigation showed that normal brain tissue (non-tumor) exhibited no or very low intensity DSE staining, and western blots of normal mouse brain tissue also revealed low expression of DSE. In contrast, there was DSE protein expression in a great portion of the glioma tissue and cell lines, and elevated DSE expression is associated with poor survival. These results indicate that other types of cancer involve elevated DSE expression, and suggest that DSE may participate in the progression of glioma.

CS/DS hybrid chains have greater structural flexibility than pure CS chains, and have greater affinity for various heparin-binding growth factors [23, 32, 33]. For instance, IdoA-containing domains interact with fibroblast growth factor 7 (FGF7) and hepatocyte growth factor (HGF) [34, 35]. These growth factor pathways are important for tumor development and progression. However, the direct biological functions of DSE and CS/DS chains in cancer cells were not investigated until chondroitin–glucuronate C5 epimerase activity was reported in 2006 [36]. It has been proposed that DSE promotes DS formation, resulting in enhanced HGF binding to the cell surface and the activation of downstream signaling in esophagus cancer cells [21]. Our results also indicated that DSE modulates DS chain formation in glioma cells. Importantly, we report for the first time that DSE selectively regulates HB-EGF-induced signaling, but not EGF and NRG1, implying that changes to DS could modulate the activity of HB-EGF or its affinity for EGFR/ErbB2 receptors. In addition, it would be worth to further investigate whether HB-EGF and HGF signaling pathways cooperate to promote DSE-mediated glioma malignancy.

HB-EGF mRNA expression is two- to five-fold higher in human glioblastomas than in normal brain tissue [37], and the HB-EGF receptors—EGFR homodimer and EGFR/ErbB2 heterodimer—are both commonly amplified in glioblastomas [29, 38, 39]. Furthermore, HB-EGF is expressed with EGFR in approximately 40% of human glioma tissues [40]. A recent study demonstrated that HB-EGF drives glioma tumorgenesis in mice lacking Ink4a/Arf and Pten through EGFR signaling [41]. These findings suggest that HB-EGF and EGFR/ErbB2 are promising therapeutic targets for glioblastoma treatment. Our data show that DSE regulates EGFR/ErbB2 signaling, and DSE expression may be positively associated with ErbB2 levels in glioblastoma cells. Although the mechanisms underlying the regulation of ErbB2 expression by DSE require further investigation, our inhibitor experiments suggest that EGFR/ErbB2 signaling could involve in the development of DSE-mediated malignant phenotypes in glioblastoma cells.

In conclusion, the results obtain in the present study suggest that DSE regulates the HB-EGF/ErbB pathway, which is involved in the DSE-induced malignant behavior of glioblastoma cells. The present study demonstrates a pathophysiologic role of DSE in glioblastoma cells, and elucidates the biological functions of aberrant CS/DS expression in glioma progression. Because there is no effective single treatment for unselected glioblastoma patients, targeting CS/DS chains with chondroitinase ABC or CSPG inhibitor, combined with other clinical trial agents, may provide novel strategies for treating this fatal disease.

## Supporting information

S1 Fig Kaplan-Meier plot of overall survival according to DSEL expression. The high and low expression groups are divided by median expression level of DSEL in 329 cases.
(JPG)

S2 Fig Expression of DSE in shRNA stable transfected clones. U118 cells were transfected with control shRNA plasmid or two DSE-specific shRNA plasmids. Single clones were obtained by puromycin selection for 14 days. The total protein was shown as loading control.
(JPG)

S3 Fig Subcellular localization of DSE in U118 cells. Confocal microscopy of serial scan images within a total thickness of 3.2 μm. White arrows indicate DSE (green) mainly localized at paranuclear and perinuclear cytoplasm. Scale bar, 15 μm.
(JPG)

S4 Fig Knockdown of DSE decreased cell viability in Ln18 cells. (A) Expression of DSE after siRNA transfection. Ln18 cells were transfected with non-targeting siRNA (siCon) or DSE-specific siRNA (siDSE) and analyzed at indicated time points. (B) Cell viability of Ln18 cells was analyzed by CCK8 assay. Data were represented as means ± SD from three independent experiments. *, P < 0.05; **, P < 0.01.
(JPG)

S5 Fig U118 transfectants were treated without (−)/with (+) NRG1 or EGF for 5 and 15 min. Phosphorylation levels of ERK, AKT, total ERK, and AKT were measured by western blotting.
(JPG)

## Abbreviations

CNS: central nerve system
CS: chondroitin sulfate
CSPG: chondroitin sulfate proteoglycan
DS: dermatan sulfate
ECM: extracellular matrix
GAG: glycosaminoglycan
HS: heparin sulfate
IdoA: iduronic acid
PG: proteoglycan
