# Supplementary figures and images for: DSE promotes aggressive glioma cell phenotypes by enhancing HB-EGF/ErbB signaling
Source: PLoS One. 2018 Jun 4;13(6):e0198364. doi: 10.1371/journal.pone.0198364 (PMC5986151; doi:10.1371/journal.pone.0198364)

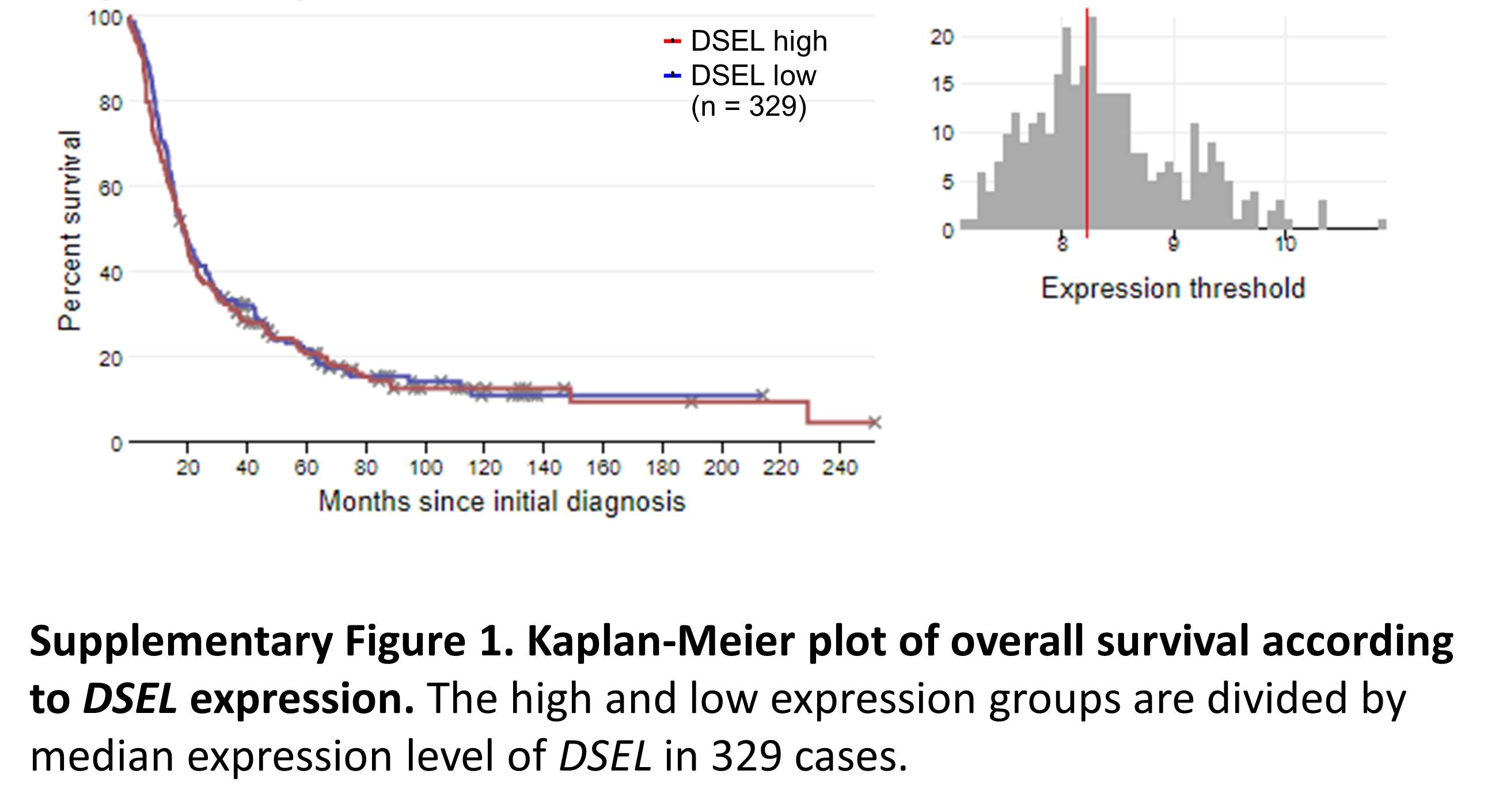

Supplement: S1 Fig — The high and low expression groups are divided by median expression level of DSEL in 329 cases. (JPG) [file pone.0198364.s001.jpg]

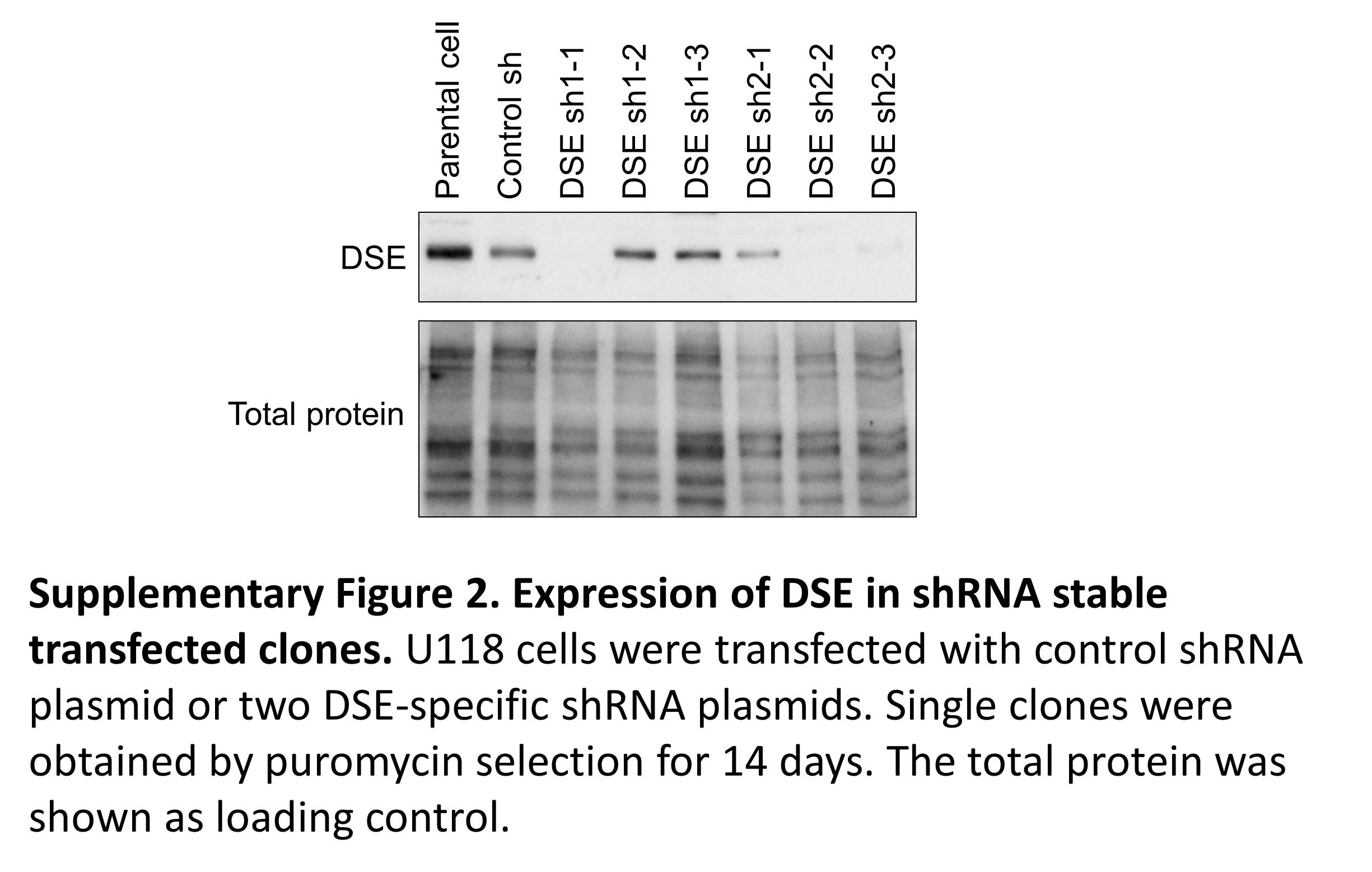

Supplement: S2 Fig — U118 cells were transfected with control shRNA plasmid or two DSE-specific shRNA plasmids. Single clones were obtained by puromycin selection for 14 days. The total protein was shown as loading control. (JPG) [file pone.0198364.s002.jpg]

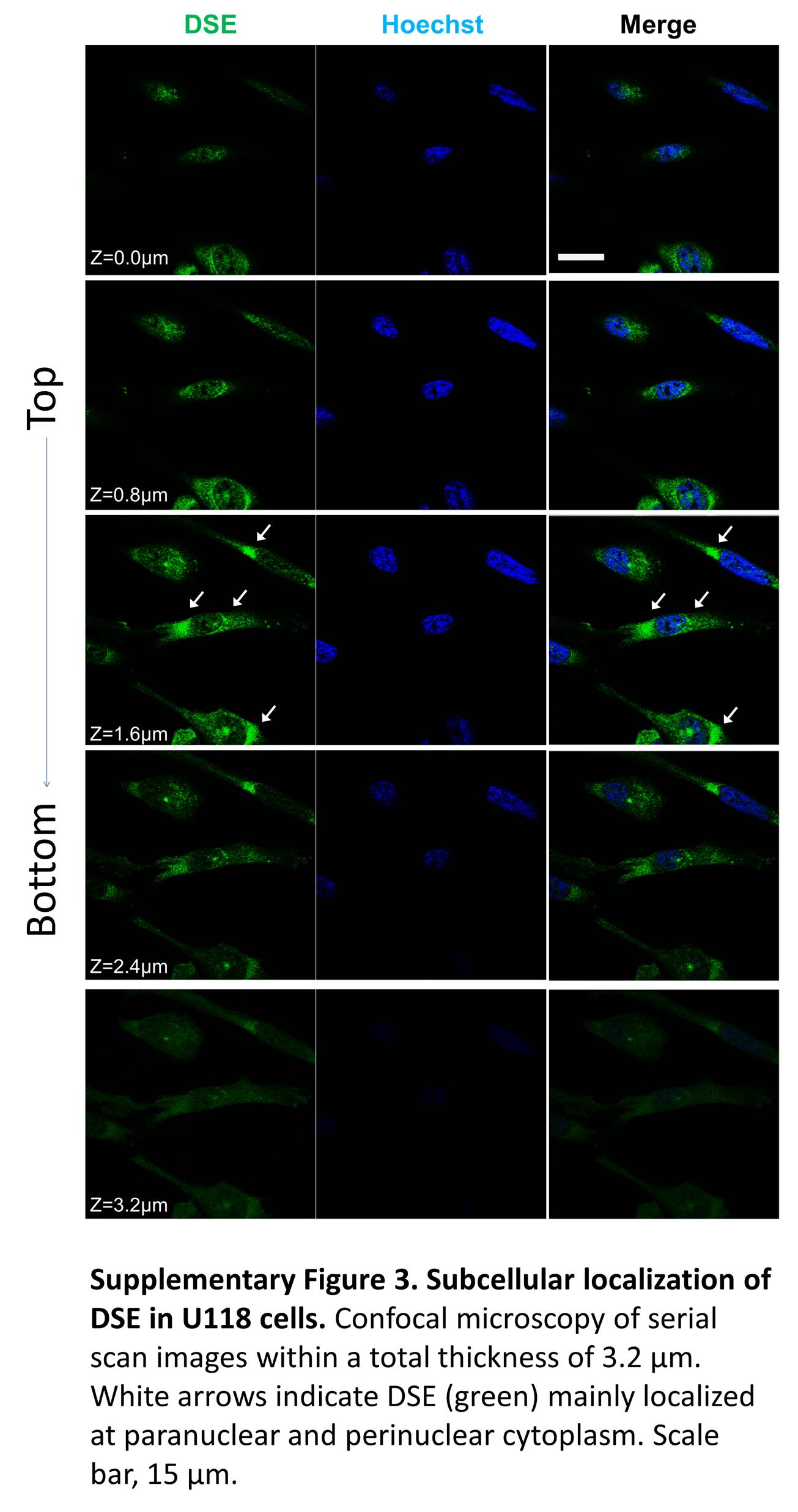

Supplement: S3 Fig — Confocal microscopy of serial scan images within a total thickness of 3.2 μm. White arrows indicate DSE (green) mainly localized at paranuclear and perinuclear cytoplasm. Scale bar, 15 μm. (JPG) [file pone.0198364.s003.jpg]

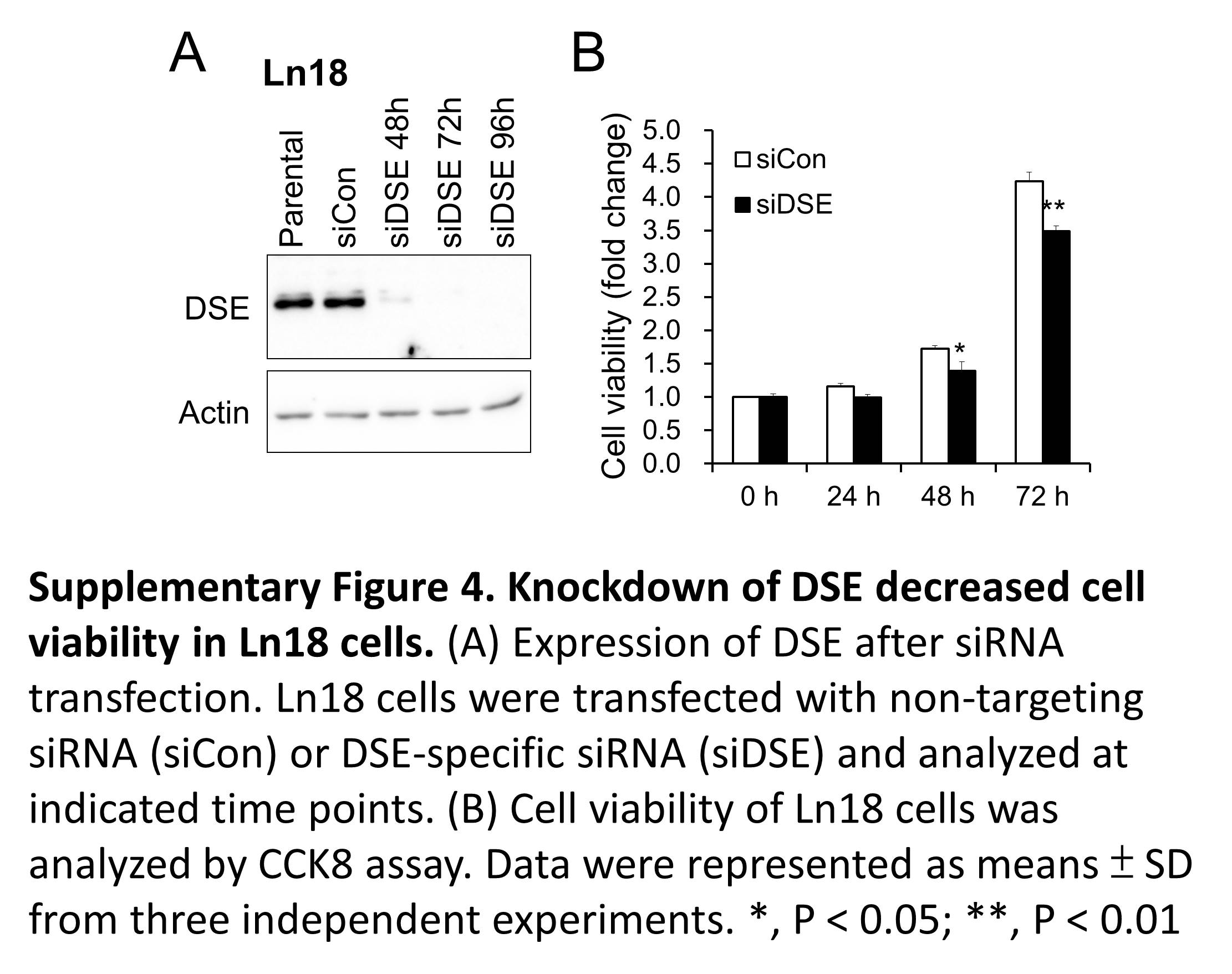

Supplement: S4 Fig — (A) Expression of DSE after siRNA transfection. Ln18 cells were transfected with non-targeting siRNA (siCon) or DSE-specific siRNA (siDSE) and analyzed at indicated time points. (B) Cell viability of Ln18 cells was analyzed by CCK8 assay. Data were represented as means ± SD from three independent experiments. *, P < 0.05; **, P < 0.01. (JPG) [file pone.0198364.s004.jpg]

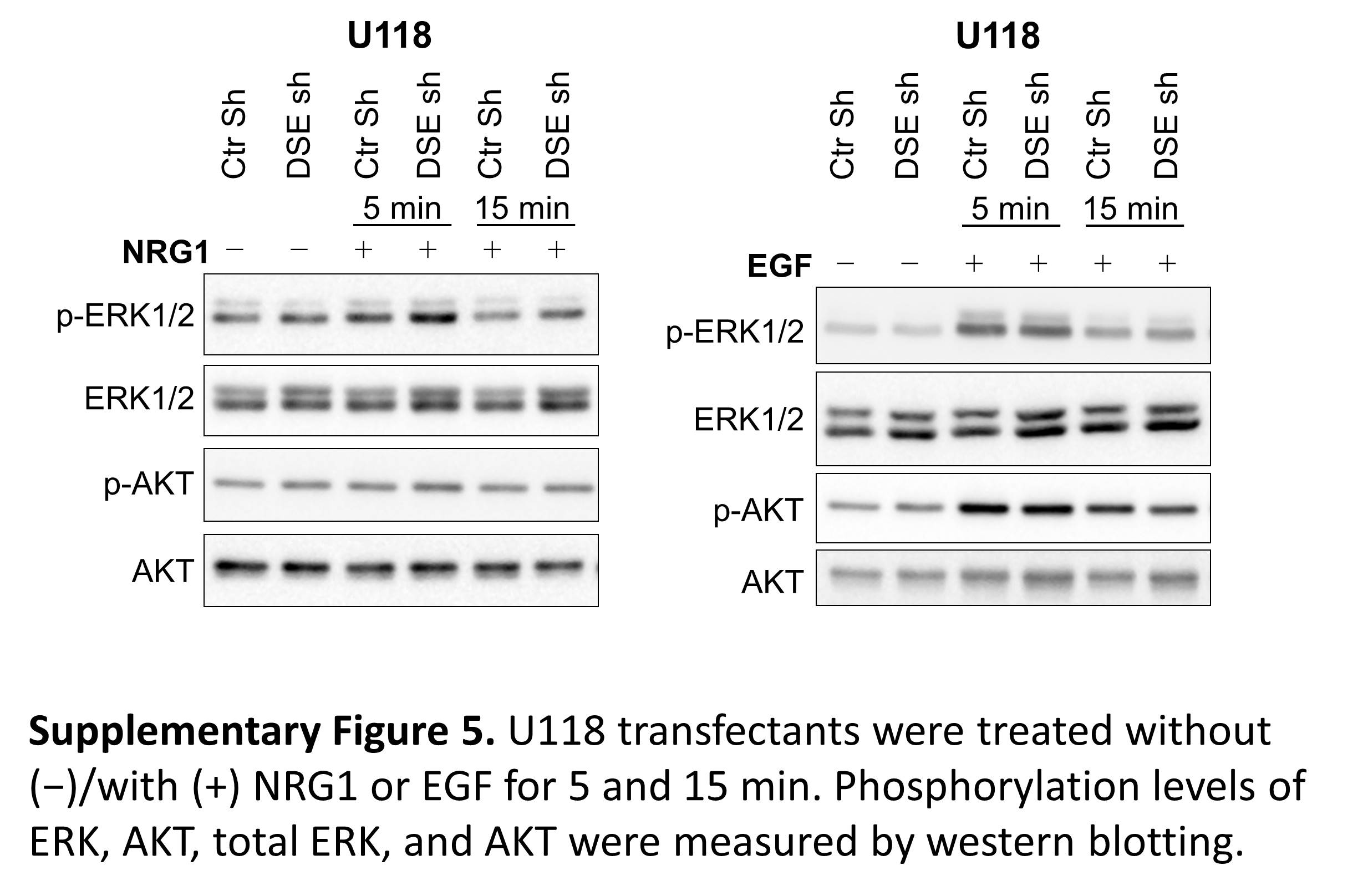

Supplement: S5 Fig — Phosphorylation levels of ERK, AKT, total ERK, and AKT were measured by western blotting. (JPG) [file pone.0198364.s005.jpg]
